# RETRACTION

**DOI:** 10.1590/1678-77572016retr001

**Published:** 2016

The Editorial Board of the Journal Applied Oral Science communicates the formal retraction of the manuscript:

ALVAREZ, Carla et al. Differential expression of CC chemokines (CCLs) and receptors (CCRs) by human T lymphocytes in response to diferente Aggregatibacter actinomycetemcomitans serotypes. J Appl Oral Sci. 2015;23(6):580-90. http://dx.doi.org/10.1590/1678-775720150285

Since it comprises a **duplicated version** of a manuscript previously published in the preceding edition of the Journal Applied Oral Science:

ALVAREZ, Carla et al. Differential expression of CC chemokines (CCLs) and receptors (CCRs) by human T lymphocytes in response to diferente Aggregatibacter actinomycetemcomitans serotypes. J App. Oral Sci. 2015;23(5):536-46. http://dx.doi.org/10.1590/1678-775720150285

Prof. Dr. Gustavo Pompermeier Garlet Editor-in-Chief

